# Influenza Virus Infection Increases Host Susceptibility To Secondary Infection with Pseudomonas aeruginosa, and This Is Attributed To Neutrophil Dysfunction through Reduced Myeloperoxidase Activity

**DOI:** 10.1128/spectrum.03655-22

**Published:** 2022-12-08

**Authors:** Feilong Jie, Xiaofeng Wu, Fan Zhang, Jiashun Li, Zijian Liu, Yizi He, Chufang Li, Hao Zhang, Yanqin Lin, Xiulong Zhu, Haijian Yu, Yichu Liu, Zhixia Li, Linbing Qu, Ling Chen, Pingchao Li

**Affiliations:** a State Key Laboratory of Respiratory Disease, Institute of Respiratory Health, The First Affiliated Hospital of Guangzhou Medical University, Guangzhou, China; b State Key Laboratory of Respiratory Disease, Guangzhou Institutes of Biomedicine and Health, Chinese Academy of Sciences, Guangzhou, China; c Department of Respiratory Medicine, Gaozhou People’s Hospital, Gaozhou, China; d Department of Respiratory Medicine, Huadu People’s Hospital, Guangzhou, China; e Guangzhou Laboratory, Guangzhou, China; f University of Chinese Academy of Science, Beijing, China; University of California, San Diego

**Keywords:** influenza virus, *P. aeruginosa*, susceptibility, neutrophil, myeloperoxidase

## Abstract

Secondary bacterial infection greatly increased the morbidity and mortality of influenza virus infection. To investigate the underlying mechanism by which influenza impairs the pulmonary defense against secondary Pseudomonas aeruginosa (P. aeruginosa) infection, we established a lethal mouse model in which to study secondary P. aeruginosa infection after influenza virus infection. We found a significant increase in host susceptibility to a secondary infection with P. aeruginosa in mice after an influenza virus infection, and this was accompanied by severe immunopathology and pulmonary inflammation. Importantly, we demonstrated that neutrophils were essential for P. aeruginosa clearance in secondarily infected mice. Further, we revealed that influenza impaired the phagocytosis and digestion functions of pulmonary neutrophils for P. aeruginosa clearance. We identified that the activity of reactive oxygen species (ROS) and the myeloperoxidase (MPO) activity of neutrophils in the lungs played an important role in antibacterial host defense in influenza-infected lungs. Hereby, influenza virus infection causes deficient MPO activity in neutrophils, and this contributes to the increased susceptibility to secondary P. aeruginosa infection. Treatment with Bacillus Calmette-Guerin polysaccharide nucleic acid (BCG-PSN) prior to secondary P. aeruginosa infection may improve the function of neutrophils, resulting in significantly reduced lethality during secondary P. aeruginosa infection. We also demonstrated that treatment with anti-influenza immune serum during the early stage of an influenza virus infection could decrease the disease severity of secondary P. aeruginosa infection. Our findings suggest that improving the MPO activity of neutrophils may provide a therapeutic strategy for viral-bacterial coinfection.

**IMPORTANCE** A secondary bacterial infection, such as that of P. aeruginosa, often occurs after a pulmonary virus infection and contributes to severe disease. However, the underlying mechanisms responsible for viral-bacterial synergy in the lung remain largely unknown. In this study, we reported that influenza virus infection increases a host’s susceptibility to secondary infection by P. aeruginosa by reducing the MPO activity of neutrophils. We also demonstrated that treatment with BCG-PSN or anti-influenza immune serum prior to secondary P. aeruginosa infection can reduce the disease severity. Our findings suggest that improving the MPO activity of neutrophils may provide a therapeutic strategy for viral-bacterial coinfection.

## INTRODUCTION

As one of the most prevalent respiratory pathogens, the influenza virus causes millions of cases of severe illnesses worldwide annually, and it results in thousands of deaths, especially in young children and elderly patients (1). Laboratory, clinical, and epidemiological studies have shown that secondary bacterial infections significantly increase the morbidity and mortality of influenza virus infections, especially in high-risk individuals, such as the immunocompromised and immunosuppressed (2, 3). It is reported that bacterial pneumonia is the predominant cause of death in pandemic influenza. During the influenza pandemic in 1918, secondary bacterial infection was the predominant cause of death in infected patients, and almost all associated deaths had at least one bacterial pathogen isolated from their respiratory tracts (4). In recent years, an increasing role of secondary infections with bacteria, especially antibiotic-resistant bacteria, has been appreciated as the cause of influenza-related mortality during seasonal influenza epidemics (5, 6).

P. aeruginosa is an opportunistic bacterial pathogen that is capable of thriving in highly diverse environments and causing significant mortality and morbidity among immunocompromised patients (7, 8). The extraordinary ability of this bacterium to become resistant to nearly all antibiotics via selected chromosomal genetic mutation and the spread of acquired resistance complicates the treatment of infected individuals (9). Gram-positive bacteria, such as Staphylococcus aureus, Streptococcus pneumoniae, and Haemophilus influenzae are known to cause post-influenza pneumonia (10), but Gram-negative bacteria, such as P. aeruginosa, are the more prominent pathogens that cause secondary bacterial pneumonia, according to recent clinical observations (2, 4). A previous study showed that coinfection with influenza virus and P. aeruginosa occurred in 8% of patients at ICU admission (11). Another study showed that P. aeruginosa infection occurred in 16.8% (180/1,087) of patients with a laboratory-confirmed viral-bacterial coinfection (2). Therefore, there is increasing emphasis on understanding the underlying immune mechanisms that increase host susceptibility to secondary bacterial pneumonia following an influenza virus infection.

As an important part of the innate immune system, neutrophils provide the first line of defense against bacterial infections (12). Neutrophils are produced and recruited into tissues within several hours after a bacterial infection, and they promptly attack and clear bacteria via phagocytosis and digestion (12). Fatal infection can be induced by the inhalation of a small amount of P. aeruginosa in the absence of neutrophils, indicating that neutrophils are essential in the host resistance against a P. aeruginosa infection (13). Thus, neutrophil dysfunction in the host impairs bacterial clearance and contributes to tissue damage by releasing dangerous cargo (14). However, the immune mechanism by which an influenza virus infection impairs neutrophil function remains unclear. In this study, we established a mouse model of a post-influenza virus, secondary P. aeruginosa infection, and we explored the mechanism by which influenza virus damages the host immune response against P. aeruginosa. Furthermore, we examined potential immunotherapy approaches by which to restore immune responses that were impaired by an influenza virus infection.

## RESULTS

### A post-influenza virus, secondary P. aeruginosa infection resulted in increased morbidity and mortality.

**FIG 1:**
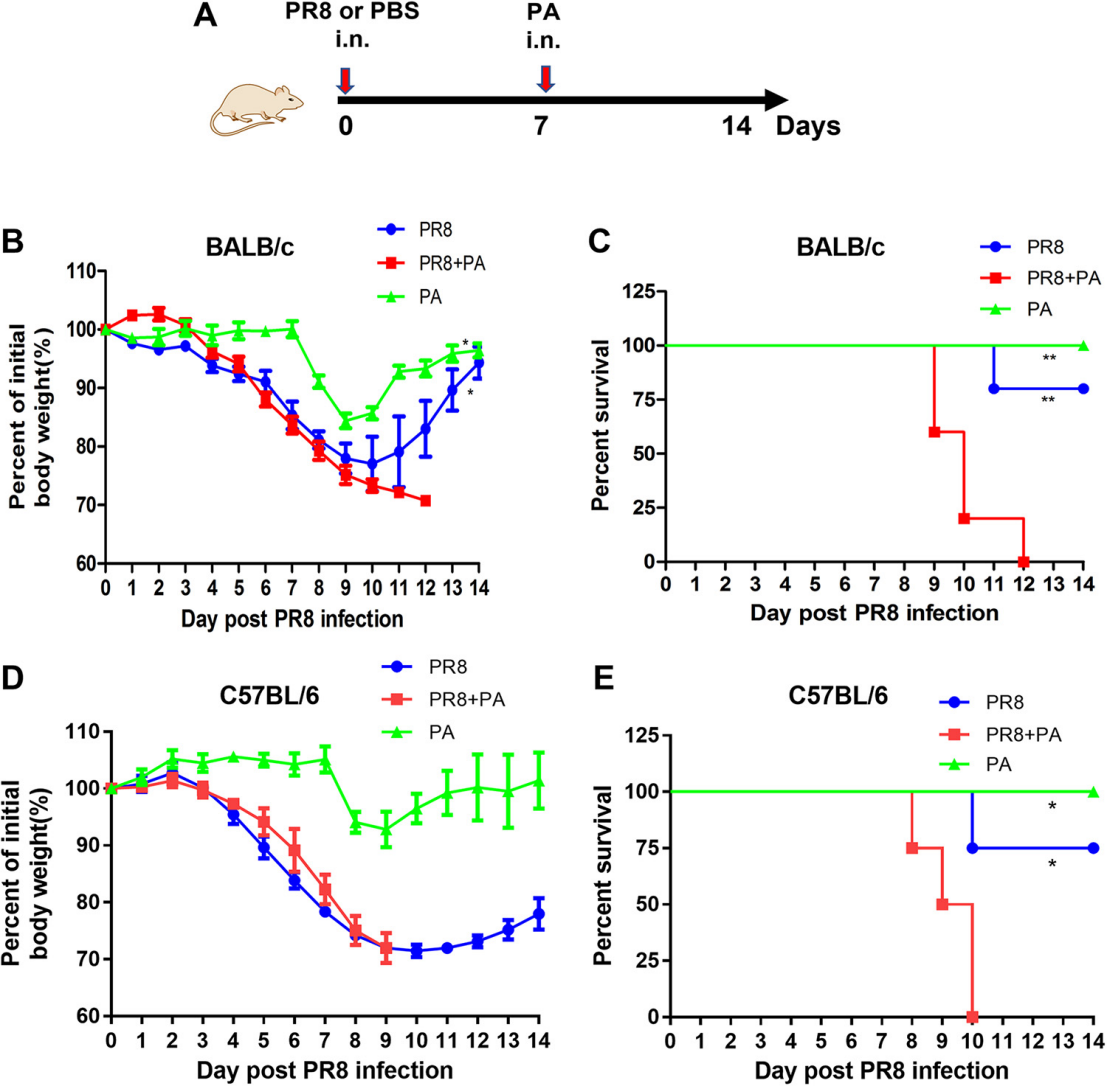
Influenza pneumonia enhances host susceptibility to secondary infection with P. aeruginosa. (A) Schematic diagram of the timeline for infection. BALB/c or C57BL/6 mice were infected with PR8 influenza virus or 40 μL PBS at day 0 and were challenged with 2 × 10^6^ CFU P. aeruginosa (PA) on day 7. The body weight loss percentage of the initial body weight (B) and the survival rate of the mice (C) were measured during infection in BALB/c mice (*n* = 5). The body weight loss percentage of the initial body weight (D) and the survival rate of the mice (E) were measured during infection in C57BL/6 mice (*n* = 4).

To mimic clinical events in which an influenza virus infection predisposes an individual to P. aeruginosa, we used a mouse-adapted influenza A virus (PR8) and the P. aeruginosa strain PA8788 (PA, P. aeruginosa) isolated from a patient in Guangzhou. Previous studies indicated that secondary infections with bacteria occur, in both humans and mice, mostly about 7 days after an influenza virus infection, at the time of influenza virus clearance (15). Based on previously reported modes of secondary P. aeruginosa infection (16, 17), we established a secondary infection model in which BALB/c mice were infected with P. aeruginosa (2 × 10^6^ colony forming units [CFU]) after 7 days following a PR8 influenza virus (20 PFU) infection via the intranasal route (Fig. 1A). Daily monitoring of the changes in the body weights of the mice reflected the overall disease severity. Mice receiving the PR8 influenza virus or P. aeruginosa alone exhibited a mild loss of body weight throughout the infection and gradually recovered in the following days (Fig. 1B). In contrast, secondarily P. aeruginosa infected mice exhibited increased morbidity as early as 9 days post-PR8 influenza virus infection (i.e., 2 days post-P. aeruginosa infection) and displayed severe losses of body weight (Fig. 1B). The secondarily P. aeruginosa infected mice also exhibited significantly increased mortality rates (100%) at 12 days post-PR8 influenza virus infection (i.e., 5 days post-P. aeruginosa infection) (Fig. 1C). To further validate this secondary infection model, we also established a secondary infection model in which C57BL/6 mice were infected with P. aeruginosa (2 × 10^6^ CFU) 7 days after a PR8 influenza virus (100 PFU) infection via the intranasal route. Consistent with the observations in the BALB/c mouse model, the secondarily P. aeruginosa infected C57BL/6 mice showed a marked loss in body weight and a higher mortality rate (100%) on day 10 post-RR8 infection (i.e., 3 days post-P. aeruginosa infection) (Fig. 1D and E). These results demonstrate that influenza virus infection impairs the host defense against P. aeruginosa infection and reduces survival in mice.

### Post-influenza virus, secondary P. aeruginosa infection increased inflammatory cytokines and caused more severe lung damage.

**FIG 2:**
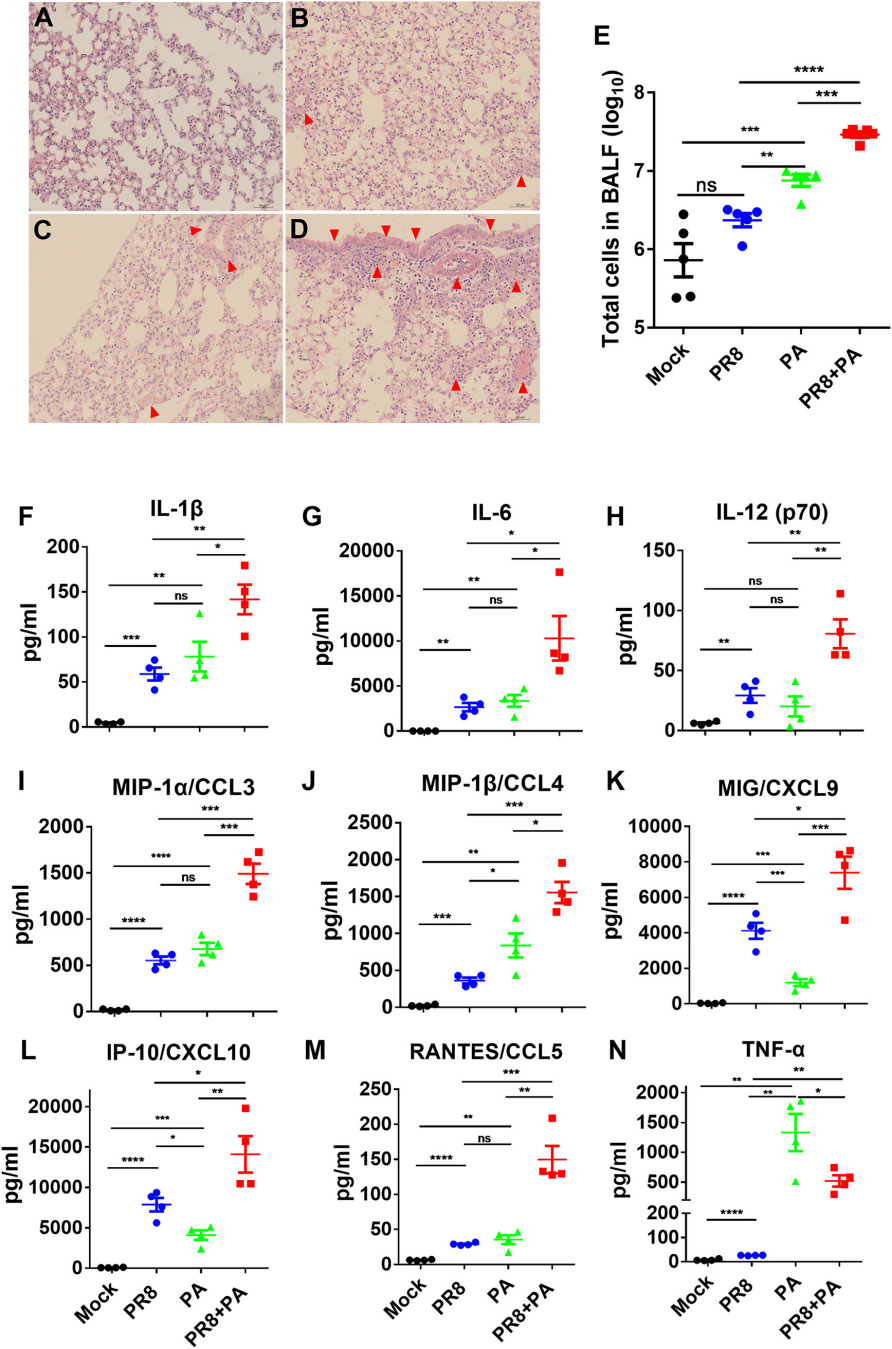
Post-influenza virus secondary P. aeruginosa infection causes more inflammation cytokines and severe lung damage. (A–D) At 24 h after P. aeruginosa (PA) infection, lung tissues from mice infected with secondary P. aeruginosa, PR8 influenza virus alone, or P. aeruginosa alone were collected and stained by hematoxylin-eosin. (A) Mock. (B) PR8 influenza virus infection alone. (C) P. aeruginosa infection alone. (D) Post-PR8 influenza virus infection secondary P. aeruginosa infection. Red arrows indicate inflammatory cell infiltrations and alveolar damage. (E) BALF from mice infected with P. aeruginosa for 24 h. Then, BALF cells were counted via flow cytometry (*n* = 5). (F–N) BALF from mice infected with P. aeruginosa for 12 h. Then, BALF supernatants were collected (*n* = 4). A panel of cytokines/chemokines, including IL-1β (F), IL-6 (G), IL-12(p70) (H), MIP-1α/CCL3 (I), MIP-1β/CCL4 (J), MIG/CXCL9 (K), IP-10/CXCL10 (L), RANTES/CCL5 (M), and TNF-α (N) were measured using a Millipore MAP Kit. Mice received PBS as a negative control (mock). *, *P* < 0.05; **, *P* < 0.01; ***, *P* < 0.001; ns, no significance.

To examine the histopathological changes in secondarily infected mice, lungs were harvested, processed, and stained with hematoxylin and eosin (H&E) at 8 days post-PR8 influenza virus infection (i.e., 1 day post-P. aeruginosa infection). Secondary P. aeruginosa infection caused substantially more lung damage than did PR8 influenza virus or P. aeruginosa infection alone. In addition, secondarily P. aeruginosa infected mice exhibited more inflammatory cell infiltrations and diffuse alveolar damage with intra-alveolar protein and/or fibrin extravasation (Fig. 2A–D). Furthermore, the total cells in the bronchoalveolar lavage fluid (BALF) of mice were counted. Consistent with the histopathological findings, the numbers of total cells in the BALF were significantly higher in the secondary P. aeruginosa infected mice, compared to mice infected with PR8 influenza virus or P. aeruginosa alone (Fig. 2E).

To further evaluate the underlying mechanisms of lung damage in the secondarily P. aeruginosa infected mice, inflammatory cytokines in the BALF samples of mice were measured via a Luminex assay at 12 h after a P. aeruginosa infection. The levels of IL-1β, IL-6, IL-12 (p70), MIP-1α/CCL3, MIP-1β/CCL4, MIG/CXCL9, IP-10/CXCL10, and RANTES/CCL5 were significantly increased in secondarily P. aeruginosa infected mice, compared to mice infected with PR8 influenza virus or P. aeruginosa alone (Fig. 2F–M). On the other hand, TNF-α, which can induce neutrophil adhesion, was significantly decreased in secondarily P. aeruginosa infected mice, compared with mice infected with P. aeruginosa alone (Fig. 2N). These results suggest that the secondary P. aeruginosa pneumonia that followed a PR8 influenza virus infection resulted in a significant increase in inflammatory cytokines that led to a cytokine storm, which contributed to severe lung damage.

### Neutrophils play an important role in P. aeruginosa clearance in secondarily infected mice.

**FIG 3:**
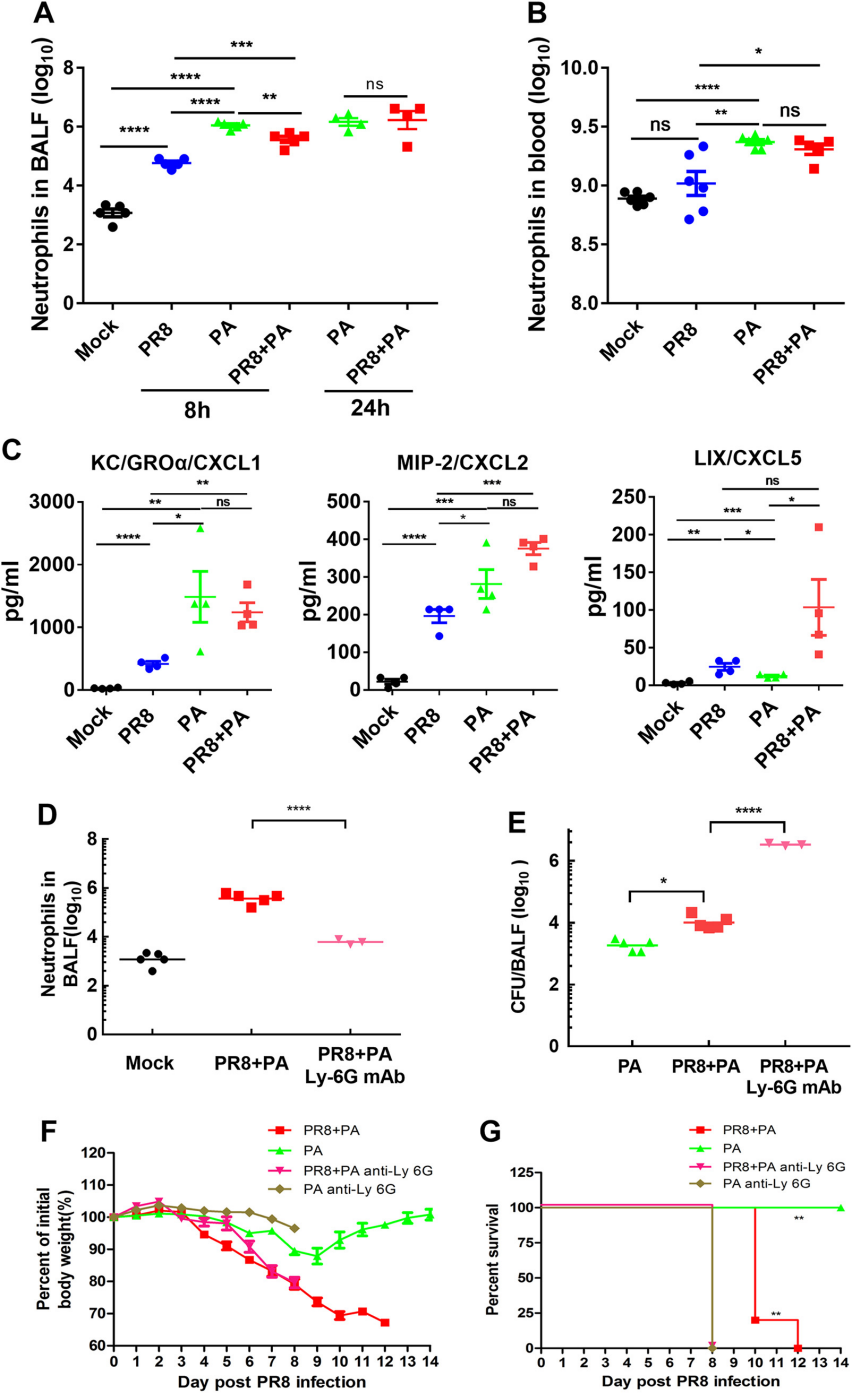
The recruitment rate of neutrophils and clearance of P. aeruginosa are diminished in secondarily infected mice. (A–C) BALB/c mice were infected with 20 PFU of PR8 influenza virus or 40 μL PBS (mock) for 7 days and were then challenged with 2 × 10^6^ CFU P. aeruginosa (PA). At different time points after PA infection, the mice were sacrificed, and BALF was collected. (A) The number of neutrophils in the BALF at 8 h and 24 h after P. aeruginosa infection was counted via flow cytometry (*n* = 4 to 5). (B) The number of neutrophils in the peripheral blood at 24 h after P. aeruginosa infection were counted via flow cytometry (*n* = 6). (C) KC/CXCL1, MIP-2/CXCL2, and LIX/CXCL5 were measured in the BALF supernatants at 12 h after P. aeruginosa infection via a Luminex assay (*n* = 4). (D–G) Secondarily infected BALB/c mice were depleted of neutrophils via the intraperitoneal injection of 100 μg of anti-mouse Ly6G antibody 24 h before P. aeruginosa infection. (D) The number of neutrophils in the BALF at 8 h after P. aeruginosa infection was measured via flow cytometry (*n* = 3 to 5). (E) The bacterial burden in the BALF at 8 h after P. aeruginosa was measured (*n* = 3 to 5). The body weight loss percentage of the initial body weight (F) and the survival rate of the mice (G) were measured during infection (*n* = 5). *, *P* < 0.05; **, *P* < 0.01; ***, *P* < 0.001; ns, no significance.

One potential factor that can contribute to aggravating disease severity in secondary bacterial infected mice is an increased bacterial burden in the lung. To test this hypothesis, secondarily infected mice were sacrificed at 8 h after a P. aeruginosa infection in order to determine the bacterial burdens in the BALF (Fig. 3E). These results indicated that influenza virus infection caused impaired pulmonary P. aeruginosa clearance.

Neutrophils play an essential antimicrobial function in the first line of defense against invading pathogens. To determine how the influenza virus impairs bacterial clearance, we first examined the recruitment of airway neutrophils following an infection by the PR8 influenza virus and/or P. aeruginosa. Based on previous reports, neutrophils were defined as CD11b^+^Ly6G^+^ via flow cytometry. The results showed that significant neutrophils were recruited into the respiratory tract at 8 h after a P. aeruginosa infection, and the recruited neutrophils in mice infected with secondary P. aeruginosa were significantly lower than those in mice infected with P. aeruginosa alone (Fig. 3A). However, comparable numbers of BALF neutrophils were found in mice infected with P. aeruginosa alone and in mice infected with secondary P. aeruginosa, and these numbers were greater than those observed in mice infected with PR8 influenza virus alone at 24 h after a P. aeruginosa infection (Fig. 3A). Consistent patterns of neutrophils were also found in the peripheral blood at 24 h after a P. aeruginosa infection (Fig. 3B).

Chemokines play a crucial role in recruiting blood neutrophils to infected tissues. Neutrophil chemotaxis-associated cytokines in the BALF samples of mice were measured via a Luminex assay at 12 h after a P. aeruginosa infection. Consistent with the neutrophil numbers in the respiratory tract, KC/CXCL1 and MIP-2/CXCL2 were significantly increased in mice infected with P. aeruginosa (Fig. 3C). However, comparable levels of KC/CXCL1 and MIP-2/CXCL2 were detected in P. aeruginosa infected and secondarily P. aeruginosa infected mice, and these numbers were greater than those observed in mice infected with the PR8 influenza virus alone (Fig. 3C). Surprisingly, although pulmonary P. aeruginosa infection alone induced an increase in low levels of LIX/CXCL5, the level of LIX/CXCL5 was significantly increased in secondary P. aeruginosa infected mice, compared with mice infected with P. aeruginosa alone (Fig. 3C). This suggests that influenza virus infection impairs the rate of neutrophil recruitment into the airway.

To determine the contribution of neutrophils in the increased host susceptibility to P. aeruginosa during a PR8 influenza virus infection, we depleted the neutrophils in secondarily infected mice by intraperitoneally injecting the anti-mouse Ly6G monoclonal antibody 24 h before P. aeruginosa infection. To validate the effects of neutrophil depletion via the Ly6G antibody, we measured the number of neutrophils present in the BALF of depleted and nondepleted secondarily infected mice after 8 h of P. aeruginosa infection. The mice that received the anti-mouse Ly6G antibody had an approximately 100-fold reduction in BALF neutrophils, compared to the nondepleted mice, indicating that the BALF neutrophils can be effectively depleted by the anti-mouse Ly6G antibody in secondarily P. aeruginosa infected mice (Fig. 3D). Neutrophil-depleted, secondarily infected mice had more than 100-fold higher P. aeruginosa CFU in the BALF at 8 h after P. aeruginosa infection than did the nondepleted mice (Fig. 3E). The neutrophil-depleted mice showed an increased host susceptibility to P. aeruginosa alone and to a secondary P. aeruginosa infection that followed a PR8 influenza virus infection, as reflected by the increased disease severity and decreased survival rates, compared to either those of mice infected with P. aeruginosa alone or those of secondarily P. aeruginosa infected mice (Fig. 3F and G). Together, these data suggest that BALF neutrophils play a crucial role in the increased host susceptibility to P. aeruginosa infection.

### Influenza virus infection impaired phagocytosis and the digestion functions of pulmonary neutrophils.

**FIG 4:**
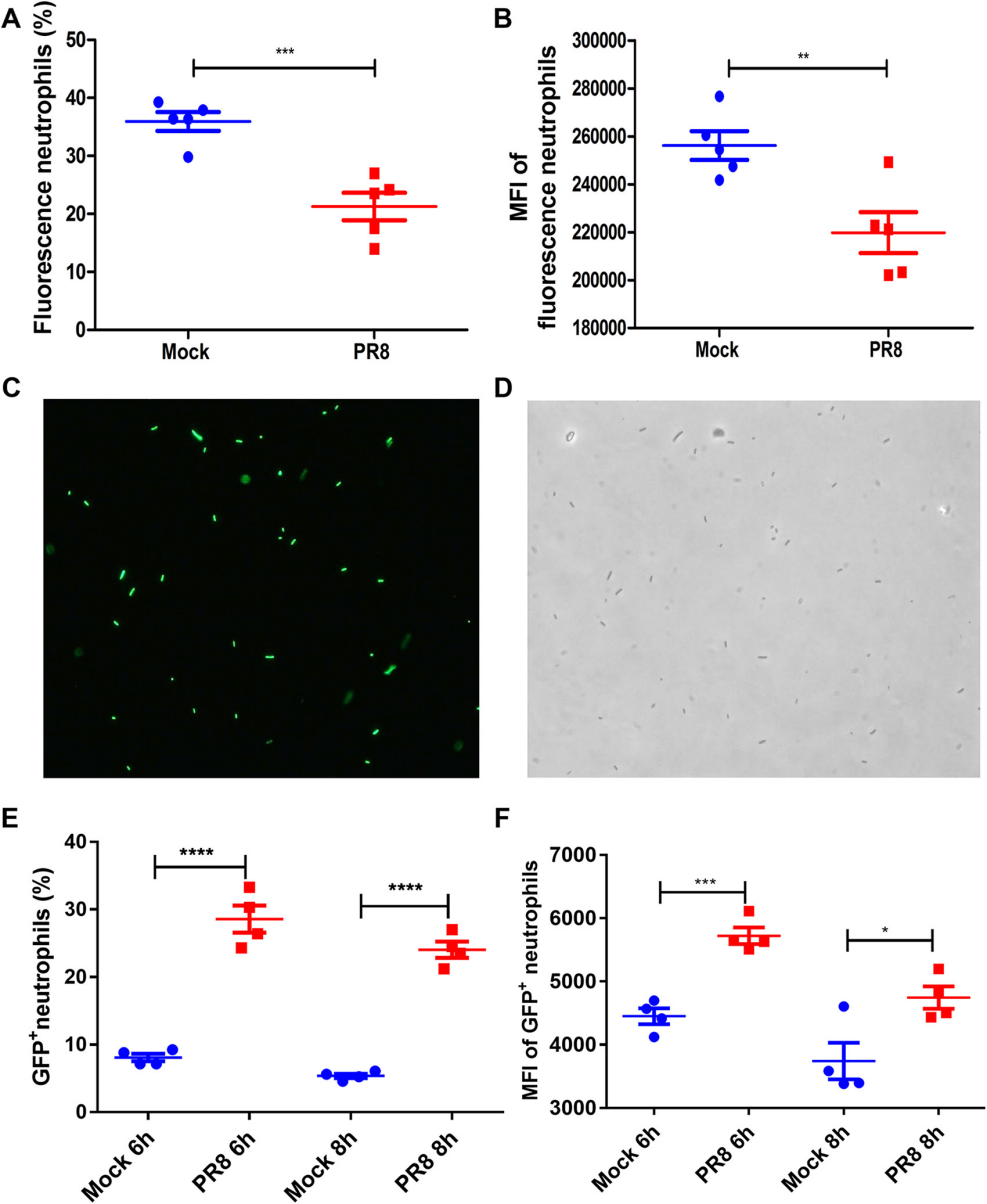
Phagocytosis and digestion of pulmonary neutrophils are impaired by influenza virus infection. (A and B) BALB/c mice were infected with 20 PFU of PR8 influenza virus or 40 μL PBS (mock) for 7 days and then challenged with a mixture of 2 × 10^6^ CFU P. aeruginosa and 2 × 10^7^ PFU Fluorescent YG microspheres. At 8 h after bacterial infection, the mice were sacrificed, and BALF was collected. The percent (A) and mean fluorescent intensity (MFI) (B) of fluorescence-positive neutrophils in the BALF were measured via flow cytometry (*n* = 5). (C and D) GFP-labeled *E. coli* (GFP-*Top10*) was constructed via transformation with the plasmid pGFPuv into *E. coli*. The labeling rate of GFP in GFP-*Top10* was measured via fluorescence microscopy. (E and F) Mice were infected with PR8 influenza virus or PBS (mock) for 7 days, and this was followed by infection with 2 × 10^7^ PFU of GFP-*Top10*. BALF was collected at 6 h and 8 h after P. aeruginosa infection. The percent (E) and MFI (F) of GFP-positive neutrophils in the BALF were measured via flow cytometry (*n* = 4). *, *P* < 0.05; **, *P* < 0.01; ***, *P* < 0.001; ns, no significance.

To determine whether influenza virus infections lead to defects in neutrophil phagocytosis *in vivo*, the phagocytosis of Fluorescent YG microspheres via pulmonary neutrophils in the mice was analyzed via flow cytometry. The percentage of phagocytic neutrophils and the phagocytic ability of each cell were determined by the percentage of fluorescence (FITC) neutrophils and the fluorescence intensity of the neutrophils. Mice were infected with the influenza virus or with PBS (mock) for 7 days, and this was followed by an infection with a mixture of 2 × 10^6^ CFU of P. aeruginosa and 2 × 10^7^ PFU of Fluorescent YG microspheres. After 8 h, BALF neutrophils were collected and analyzed via flow cytometry. There was a significant decrease in the percentage of fluorescence-positive neutrophils and in the fluorescence intensity of the neutrophils after PR8 influenza virus infection, compared to mice without PR8 influenza virus infection (Fig. 4A and B). This showed that a prior influenza virus infection decreases the phagocytotic abilities of pulmonary neutrophils against P. aeruginosa.

Next, we evaluated whether the influenza virus affected the digestion of bacteria by neutrophils. GFP-labeled E. coli (GFP-*Top10*) was constructed via transformation with the plasmid pGFPuv into *E. coli*. The GFP signal from GFP-*Top10* can be detected by fluorescence microscopy, indicating that it can be used for the assessment of neutrophil digestion (Fig. 4C and D). Mice were infected with influenza virus or PBS (mock) for 7 days, and this was followed by infection with 2 × 10^7^ PFU of GFP-*Top10*. After 6 h and 8 h, BALF neutrophils were collected and analyzed via flow cytometry. The results showed that the percentage of fluorescence-positive neutrophils and the fluorescence intensity of neutrophils in mice infected with the PR8 influenza virus were significantly increased, compared to those in PR8 influenza virus-infected mice (Fig. 4E and F). These results revealed that the neutrophils in mice infected with an influenza virus are impaired in terms of killing bacteria, although these neutrophils could phagocytose bacteria.

### Influenza virus infection impaired the ROS and MPO activities of pulmonary neutrophils.

**FIG 5:**
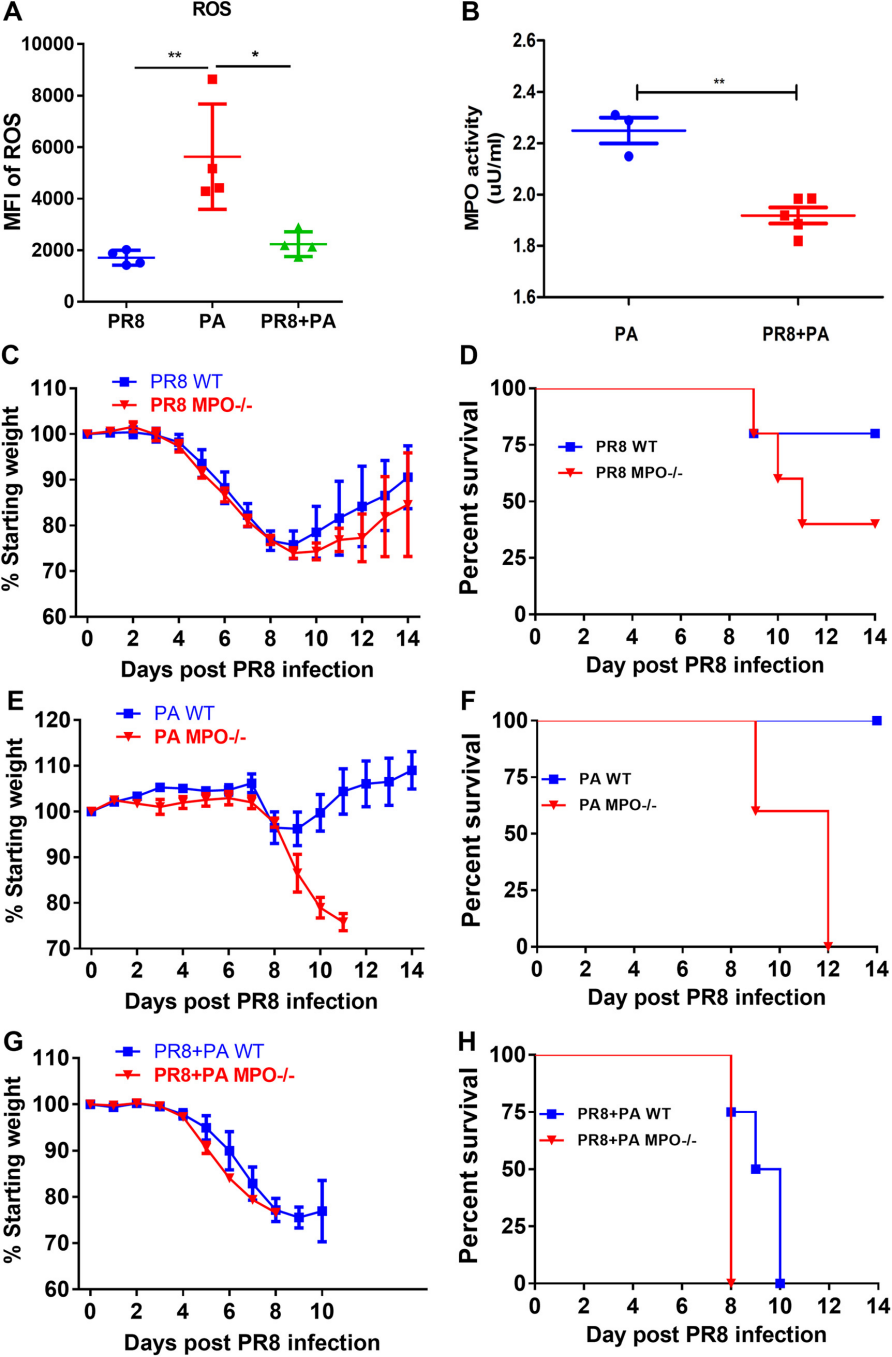
The ROS and MPO activity of pulmonary neutrophils are impaired by influenza virus infection. (A) At 24 h after P. aeruginosa (PA) infection, secondarily infected mice were sacrificed, and BALF was collected. The ROS production in neutrophils was measured via the incubation of the DCFH-DA probe. The mean fluorescent intensity (MFI) of probe-positive neutrophils in the BALF was measured via flow cytometry (*n* = 4). (B) At 8 h after P. aeruginosa infection, the mice were sacrificed, and BALF was collected. The MPO activity in BALF cells was measured using an MPO activity colorimetric assay kit (*n* = 3 to 5). (C–H) (*n* = 4 to 5). WT and MPO-deficient C57BL/6 mice were infected with PR8 influenza virus alone, infected with P. aeruginosa alone, or secondarily infected with PR8 influenza virus and P. aeruginosa. The body weight loss percentage of the initial body weight (C) and the survival rate of the mice (D) were measured in PR8 influenza virus-infected mice. The body weight loss percentage of the initial body weight (E) and the survival rate of mice (F) were measured in P. aeruginosa-infected mice. The body weight loss percentage of the initial body weight (G) and the survival rate of the mice (H) were measured in secondarily infected mice. *, *P* < 0.05; **, *P* < 0.01; ***, *P* < 0.001; ns, no significance.

ROS produced by neutrophils play an important role in killing invading microbes (18). Next, we determined whether the phagocyte oxidative burst was impaired following an influenza virus infection. BALF cells from mice were labeled with a 2,7-dichlorodihydrofluorescein diacetate (DCFH-DA) probe, and the fluorescence intensity of the neutrophils was analyzed via flow cytometry. Significantly decreased ROS levels were observed in neutrophils obtained from PR8 influenza virus infected or secondarily P. aeruginosa infected mice, compared with mice infected with P. aeruginosa alone (Fig. 5A). In addition, MPO, as a heme-containing peroxidase that is expressed mainly in neutrophils, catalyzed the formation of reactive oxygen intermediates. MPO plays an important role in microbial killing and in the mediation of the inflammation response of neutrophils. The MPO activity in the BALF cells was measured using an MPO activity colorimetric assay kit. The MPO activity in the BALF cells of mice infected with PR8 influenza virus was significantly decreased, compared with that of mice without PR8 influenza virus infection after 8 h of P. aeruginosa infection (Fig. 5B). We further determined whether the MPO deficiency can increase the disease severity in P. aeruginosa infected or secondarily P. aeruginosa infected mice. Consistent with the MPO activity levels, although the influenza virus infection increased the mortality rate of the MPO-deficient mice, the MPO-deficient mice were more susceptible to P. aeruginosa infection alone and to secondary P. aeruginosa infection than to PR8 influenza virus infection alone, (Fig. 5C–H). These results suggested that influenza virus infection impairs the ability of neutrophils to mediate ROS-associated and MPO-associated antibacterial functions.

### BCG-PSN administration improves survival during secondary P. aeruginosa infections.

**FIG 6:**
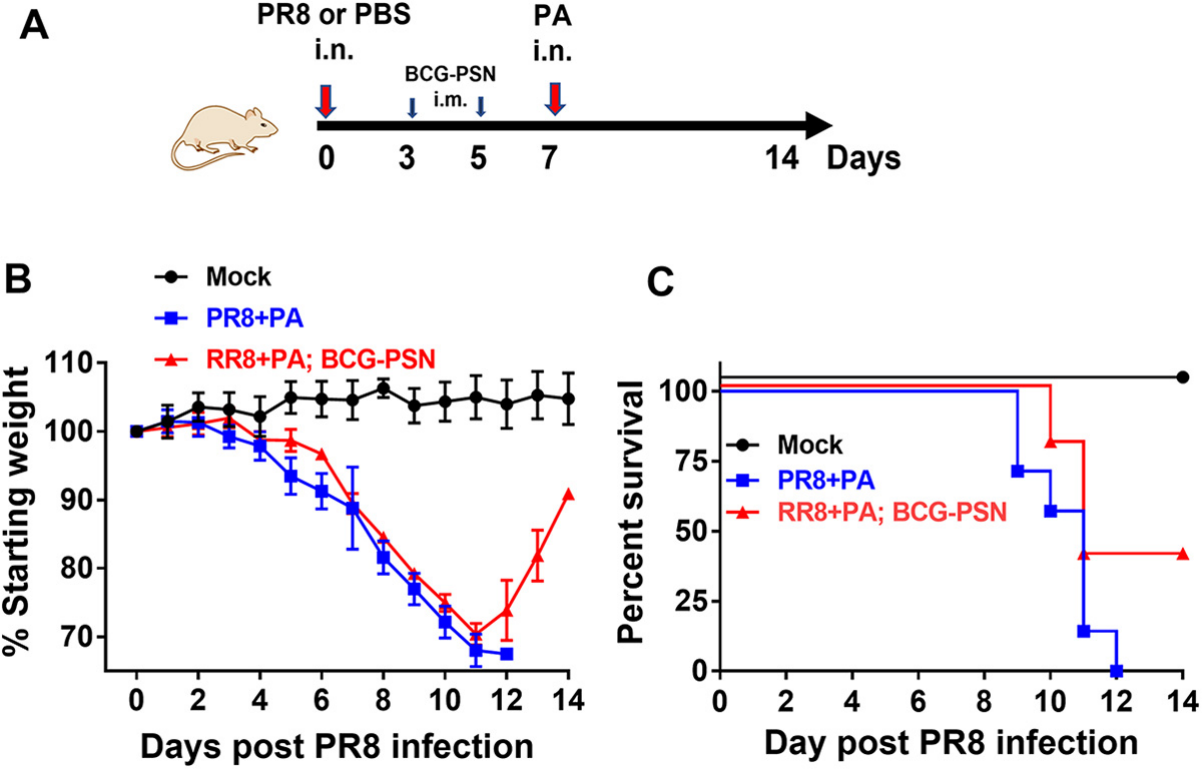
BCG-PSN administration improves survival during secondary P. aeruginosa infection. (A) Secondary P. aeruginosa (PA) infected mice were intramuscularly administered 200 μL BCG-PSN or PBS at 3 days and 5 days post-PR8 influenza virus infection, respectively. Mice received PBS only as a negative control. The body weight loss percentage of the initial body weight (B) and the survival rate of the mice (C) were measured in secondarily infected mice (*n* = 5 to 7).

Previous studies revealed that bacterial lipopolysaccharide can improve the antimicrobial function of neutrophils by eliciting multiple neutrophil responses. BCG-PSN is an immunomodulator and is a bacteria lipopolysaccharide fraction that can be extracted from Bacillus Calmette-Guerin (BCG) (19, 20). BCG-PSN has previously been used for the clinical treatment of immune deficiency disease and allergic diseases (21, 22). We determined whether the increased host susceptibility to P. aeruginosa infection could be prevented by treatment with BCG-PSN. PR8 influenza virus-infected mice were administered BCG-PSN at 3 days and 5 days post-PR8 influenza virus infection (i.e., 4 days and 2 days before P. aeruginosa infection) (Fig. 6A). We found that *in vivo* treatment with BCG-PSN did not significantly decrease the morbidity but did decrease the mortality of secondarily P. aeruginosa infected mice (Fig. 6B and C). Therefore, BCG-PSN treatment enhanced resistance to a lethal secondary P. aeruginosa infection challenge.

### Influenza-specific immune serum treatment improves survival in mice with secondary P. aeruginosa infections.

**FIG 7:**
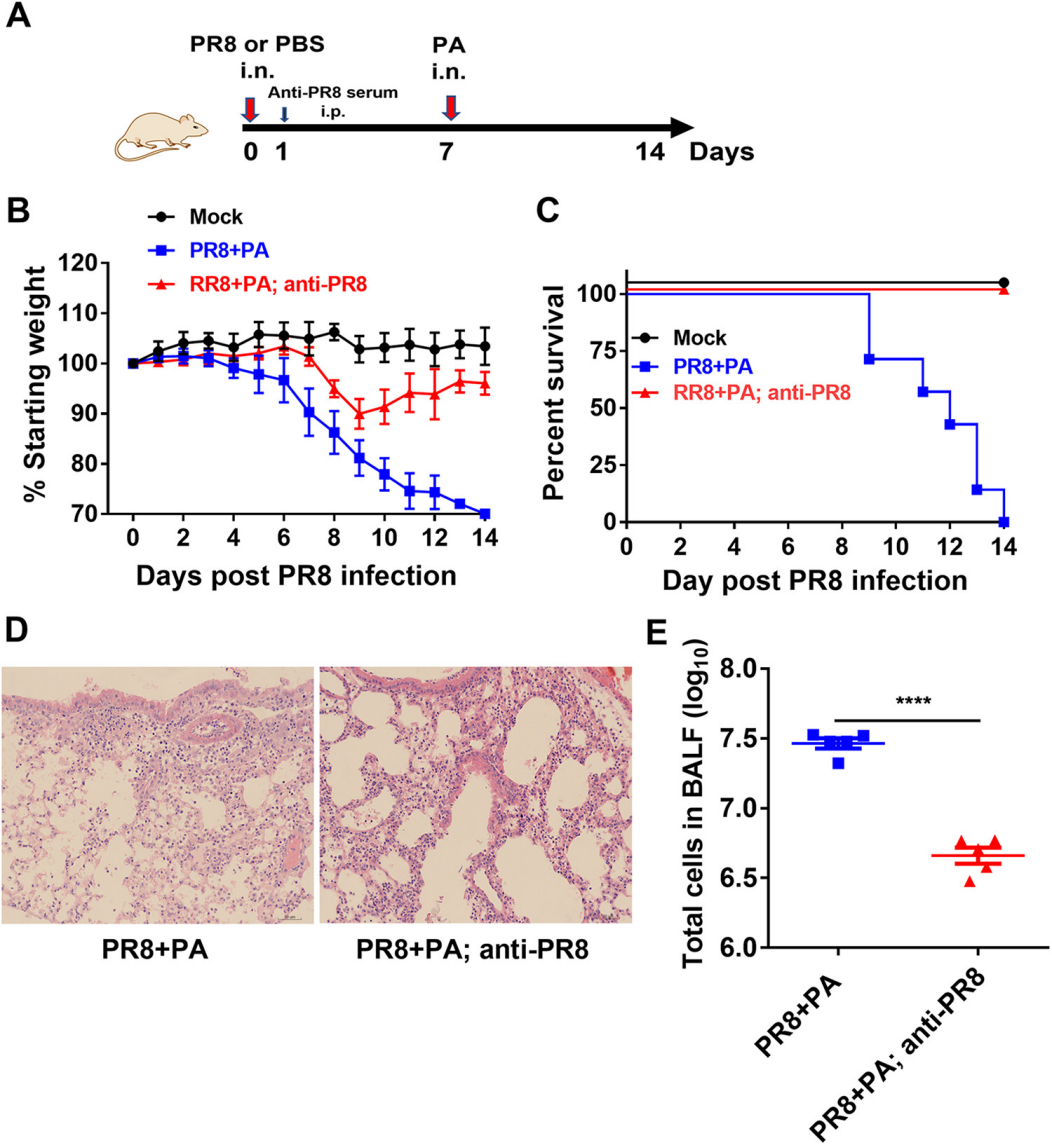
Treatment with influenza-specific immune serum improves survival in mice with a secondary P. aeruginosa infection. (A) Secondarily P. aeruginosa (PA) infected mice were intraperitoneally administered 200 μL (hemagglutination inhibition antibody titer: 200) of anti-PR8 influenza virus serum or mock serum at 1 day post-PR8 influenza virus infection. The mice received the mock serum only as a negative control. The body weight loss percentage of the initial body weight (B) and the survival rate of the mice (C) were measured in secondarily infected mice (*n* = 7). (D) At 24 h after P. aeruginosa infection, lung tissues from secondarily infected mice were collected and stained by hematoxylin-eosin. (E) The number of neutrophils in the BALF at 24 h after P. aeruginosa infection was counted via flow cytometry (*n* = 5). ***, *P* < 0.001.

We also conducted experiments to determine whether increased host susceptibility to P. aeruginosa infection could be prevented by anti-influenza serum during early infection. Secondary P. aeruginosa infected mice were intraperitoneally injected with influenza-specific immune serum on day 1 post-PR8 influenza virus infection (Fig. 7A). The injection of influenza-specific immune serum after a secondary P. aeruginosa infection decreased the disease severity and rescued 100% of the secondarily infected mice from death, whereas all of the mice receiving mock serum died (Fig. 7B and C). Lung histopathology showed that secondarily P. aeruginosa infected mice receiving the influenza-specific immune serum displayed a significant decrease in lung damage, compared to mice receiving the mock serum (Fig. 7D). Secondarily P. aeruginosa infected mice receiving the anti-influenza serum treatment also displayed a significant reduction in the total cells in the BALF, compared to untreated mice (Fig. 7E). Taken together, these results suggest that the early anti-influenza treatment was effective in decreasing the disease severity and improving the survival associated with secondary P. aeruginosa infections.

## DISCUSSION

A secondary bacterial infection after an influenza virus infection is well-recognized to cause severe pneumonia and death. In addition, it has been reported that multiple mechanisms are involved in influenza-induced host susceptibility to a subsequent bacterial infection (4, 16). Consistent with a secondary Gram-positive bacterial infection, we showed in this study that influenza virus infections decreased host antimicrobial responses to the Gram-negative bacterium P. aeruginosa, and this was reflected by a reduction of the phagocytic and digestion capacities of recruited lung neutrophils.

Neutrophils are the first inflammatory cells to be recruited to an infection site to control a microbial infection (23). Lung neutrophil recruitment is mainly mediated by chemokines, including KC/CXCL1, MIP-2/CXCL2, and LIX/CXCL5. In mice, the chemotactic effects mediate via binding to the chemokine receptors CXCR2 (23). In this study, we found a difference in the expression patterns of neutrophil-associated chemokines (KC/CXCL1, MIP-2/CXCL2, and LIX/CXCL5), most likely due to their different roles in attracting neutrophils to infected tissues. Previous study has shown that KC/CXCL1 is expressed at high levels before neutrophil infiltration, whereas LIX/CXCL5 is produced later and at lower levels, and that this occurs in parallel with neutrophil infiltration into the corneal stroma in a lipopolysaccharide (LPS)-induced keratitis mouse model (24). Furthermore, in an acute bacterial pyelonephritis mouse model, early neutrophil migration is primarily driven by KC/CXCL1, whereas neutrophil infiltration in tissue is strongly dependent on LIX/CXCL5. In addition, LIX/CXCL5 deficiency did not affect early neutrophil migration or bacterial clearance (25). In our post-PR8 influenza virus secondary P. aeruginosa infection model, we found that the level of LIX/CXCL5 was significantly increased compared with that of P. aeruginosa infection alone, implying more neutrophil infiltration into the lung parenchyma and stronger lung injuries, as confirmed by our pathological results. We speculate that lung neutrophil recruitment is mediated by KC/CXCL1 and MIP-2/CXCL2, whereas lung injury is caused by LIX/CXCL5. Thus, LIX/CXCL5-targeting therapeutics may restrict pathogenic neutrophil infiltration in the lung and may alleviate lung injury during secondary bacterial infections.

The migration of neutrophils involves a sequential, multistep adhesion cascade that is mediated by adhesion molecules, such as selectins and integrins (26). TNF-α, as a major proinflammatory cytokine that participates in early/acute inflammation, can induce neutrophil adhesion via formin-dependent cytoskeletal reorganization and via the activation of the β-integrin function in order to facilitate neutrophil migration to sites of inflammation (27, 28). In our post-PR8 influenza virus, secondary P. aeruginosa infection model, we found that the level of TNF-α was significantly decreased, compared with that of P. aeruginosa infection alone, implying slower neutrophil migration to the lung. This was confirmed by our neutrophil numbers in the BALF at 8 h after P. aeruginosa infection. Thus, increasing TNF-α in the early stages of a secondary bacterial infection may improve the migration rate of neutrophils and alleviate symptoms.

MPO is the major enzyme produced by neutrophils, and it has an antibacterial function during endotoxemia and sepsis (29, 30). A previous study suggested that diminished MPO activity may be a good predictor for identifying septic patients who are at a high risk of death (31). Furthermore, as the major source of MPO after LPS injection *in vivo*, the treatment of wild-type mice with MPO inhibitor 4-aminobenzoic acid hydrazide significantly increased mortality in response to LPS (32). In this study, we first confirmed that MPO-deficient mice have increased mortality in the post influenza virus, secondary P. aeruginosa infected mice as well as in the mice infected with P. aeruginosa alone. The protective mechanism of neutrophil-derived MPO during a P. aeruginosa infection may be intrinsically MPO-dependent and/or secondary to neutrophil phenotypic or functional alterations. It has been reported that MPO binds to neutrophils, activates neutrophil adhesion, and promotes neutrophil survival primarily via its interaction with the CD11b/CD18 integrin (33). MPO has also been reported to catalyze the oxidation and peroxidation of lipid mediators, such as leukotrienes and prostaglandins, that characterize systemic inflammation (34, 35). Thus, our findings imply that, in addition to the direct anti-P. aeruginosa function of neutrophils, neutrophil-derived MPO may contribute to optimal host protection by modulating inflammation as well as by limiting the toxic effects of P. aeruginosa.

In our post-influenza virus, secondarily P. aeruginosa infected mouse model, the increased host susceptibility was partly restored through the administration of BCG-PSN prior to a P. aeruginosa infection. However, the immune mechanism by which BCG-PSN improves the antimicrobial function remains to be further studied. The results of our study suggest that neutrophils play a crucial role in the increased host susceptibility to P. aeruginosa infection. Previous studies have shown that bacterial lipopolysaccharides can elicit a variety of neutrophil responses, including receptor upregulation, recruitment, adherence, neutrophil extracellular trap formation, and the upregulation of the NADPH oxidase, which generates superoxide and other ROS (36–40). BCG-PSN, as a bacterial lipopolysaccharide fraction that can be extracted from the BCG vaccine with a strong immunomodulatory effect (19, 22, 41), may have a similar effect on neutrophils. Recently, a study showed that BCG vaccination induced the functional reprogramming of mature neutrophils and increased the expression of activation markers as well as the antimicrobial functions of neutrophils (42). Therefore, we speculate that BCG-PSN may improve impaired neutrophil functions in our post-influenza virus, secondarily P. aeruginosa infected mice. In clinical conditions, improving neutrophil antimicrobial functions via the functional reprogramming of neutrophils may provide a new therapeutic strategy against secondary bacterial infections.

## MATERIALS AND METHODS

### Mice.

8-week-old female BALB/c and C57BL/6 mice were obtained from the Beijing Vital River Laboratory Animal Technology Co., Ltd. (Beijing, China) and housed under specific pathogen-free (SPF) conditions. The SPF, C57BL/6 MPO-deficient mice were a gift from Zhong Su from the Guangzhou Institute of Biomedicine and Health (GIBH). The animals were cared for in conformance with the animal protocol guidelines of the Committee on Animal Care of GIBH. All experiments were approved by our Institutional Animal Care and Use Committee (IACUC: 2015017).

### Influenza virus and infection.

The mouse-adapted strain of influenza virus A/Puerto Rico/8/34 (H1N1, PR8) strain was kindly provided by Weiqi Pan at GIBH. The mice were anesthetized by isoflurane, and the PR8 influenza virus was intranasally inoculated with appropriate plaque forming units (PFU) in 40 μL of PBS or with PBS as control.

### P. aeruginosa strain and infection.

The P. aeruginosa strain PA8788 (PA) used in this study was isolated from a patient in Guangzhou. The bacteria were cultured in Luria-Bertani (LB) medium at 37°C for 18 h and were then harvested via centrifugation for 2 min at 10,000 × *g*. After washing 2 times with PBS, P. aeruginosa was suspended in PBS at a concentration of 2 × 10^9^ CFU/mL, as determined by the optical density value of OD_600_ = 0.68 and confirmed by a colony forming assay. The mice were intranasally inoculated with 40 μL of PBS that contained the appropriate CFU of P. aeruginosa under anesthesia.

### Survival and body weight monitoring.

The body weights of the mice were monitored daily after PR8 influenza virus or P. aeruginosa infection. The disease severity was determined by comparing the body weights at various time points post-infection with the initial body weights pre-infection. The mice were terminated and considered dead when they lost ≥30% of their initial body weights. The mice that were dead at the time of monitoring were also recorded to determine the survival rate.

### Bronchoalveolar lavage.

The trachea was exposed through a midline incision and cannulated with a sterile 18G intravenous catheter (Vasofix, Braun). Bronchoalveolar lavage was performed by the instillation of three 0.6 mL PBS into the lung. The retrieved bronchoalveolar lavage fluids (BALF) were centrifuged at 300 × *g* for 6 min. The supernatants were collected, and the BALF cells were lysed using a red cell lysis solution. The total cell numbers were counted, and the differential cell counts were stained for flow cytometry analysis.

### Quantification of bacterial burden.

A serial 10-fold dilution of BALF in sterile PBS was plated onto LB agar plates. The plates were incubated at 37°C and 5% CO_2_, and the CFU were counted overnight.

### Histopathological analysis.

Mouse lungs were harvested at various time points post-infection, fixed in 10% formalin, and embedded in paraffin. Lung sections were stained with hematoxylin and eosin (H&E). The slides were observed and obtained using a microscope.

### Luminex assay.

For the cytokines/chemokines assay, BALF supernatants were collected from mice at 12 h after P. aeruginosa infection and were centrifuged for 6 min at 300 × *g*. The levels of IL-1β, IL-6, IL-12 (p70), MIP-1α/CCL3, MIP-1β/CCL4, MIG/CXCL9, IP-10/CXCL10, RANTES/CCL5, TNF-α, KC/CXCL1, MIP-2/CXCL2, and LIX/CXCL5 in BALF were measured using the Milliplex MAP Kit for a mouse cytokine/chemokine 96-well plate assay (Millipore, USA), following the manufacturer’s instructions.

### Flow cytometry analysis.

BALF cells were stained with the following fluorescein-labeled anti-mouse antibodies: LIVE/DEAD Aqua (Life Technology, USA), PE-eFluor 610-conjugated anti-F4/80 MAb (clone: BM8, eBioscience), eFluor 450-conjugated anti-CD11c MAb (clone: N418, eBioscience), APC-eFluor 780 conjugated anti-CD11b MAb (clone: M1/70, eBioscience), and PerCP-Cyanine5.5 conjugated anti-Ly6G MAb (clone: RB6-8C5, eBioscience) for 30 min. The strained BALF cells were detected with an LSR Fortessa flow cytometer (BD, USA). The data were analyzed using the FlowJo software package (Tree Star).

### Neutrophil depletion.

To deplete the neutrophils, mice were intraperitoneally (i.p.) injected with 100 μg of the anti-mouse Ly6G antibody (clone: 1A8-Ly6g, eBioscience, USA) at 24 h prior to the bacterial infection. Isotype IgG was injected as a control. The neutrophil depletion efficacy in BALF was confirmed via a flow cytometry analysis.

### Phagocytosis assay of neutrophils.

For the *in vivo* phagocytosis assay of neutrophils, both normal mice and influenza virus-infected mice under anesthesia were intranasally infected with 40 μL of PBS containing 2 × 10^6^ CFU P. aeruginosa and 2 × 10^7^ PFU of 1.0 μm Fluorescent YG microspheres (15702-10, Polysciences, Inc.). The bronchoalveolar lavages were performed at 6 h and 8 h post-P. aeruginosa infection, and then flow cytometry was used to assay the phagocytosis of the neutrophils.

### Evaluation of the digestion of neutrophils.

First, GFP-labeled *E. coli* was constructed via transformation with the plasmid pGFPuv into *E. coli*. After amplification, the concentration was determined via a colony forming assay and inactivation with 4% paraformaldehyde. 2 × 10^7^ CFU of GFP-labeled *E. coli* in 40 μL of PBS were intranasally inoculated to anesthetized mice. BALF cells were collected and incubated with APC-eFluor 780 conjugated anti-CD11b MAb (clone: M1/70, eBioscience) and PerCP-Cyanine5.5 conjugated anti-Ly-6G MAb (clone: RB6-8C5, eBioscience) for 30 min. The strained BALF cells were detected using an LSR Fortessa flow cytometer (BD, USA). The data were analyzed using the FlowJo software package (Tree Star, USA).

### The measurement of the reactive oxygen species of neutrophils.

The BALF cells were suspended in 200 μL of room-temperature, low sugar DMEM that contained 10% FBS with a DCFH-DA probe at a final concentration of 20 μm/L. Then, the BALF cells were transferred to 96-well plates, incubated in an incubator at 37°C and 5% CO_2_ for 30 min, and protected from light. After being washed two times with pre-cold PBS, the BALF cells were incubated with APC-eFluor 780 conjugated anti-CD11b MAb (clone: M1/70, eBioscience) and PerCP-Cyanine5.5 conjugated anti-Ly-6G MAb (clone: RB6-8C5, eBioscience) for 30 min. The strained BALF cells were detected using an LSR Fortessa flow cytometer (BD, USA). The data were analyzed using the FlowJo software package (Tree Star).

### Determination of myeloperoxidase activity in neutrophils.

To determine the myeloperoxidase (MPO) activity in the BALF cells, an MPO activity colorimetric assay kit was used for detection, according to the manufacturer’s protocol (BioVision, Milpitas, CA, USA).

### BCG-PSN or anti-influenza serum treatment.

Mice were intramuscularly administered with 200 μL Bacillus Calmette-Guerin polysaccharide nucleic acid (BCG-PSN) (Hunan Siqi Biopharmaceutical Co., Ltd.) 4 days and 2 days before P. aeruginosa infection. Mice were intraperitoneally administered with 200 μL (hemagglutination inhibition antibody titer: 200) anti-PR8 influenza virus serum at 1 day post-PR8 influenza virus infection.

### Statistical analysis.

The data were analyzed using the GraphPad Prism 7 software package. The survival of the mice was analyzed via a Kaplan-Meier analysis. Comparisons between groups were analyzed using an unpaired Student’s *t* test (two-tailed). A *P* value of <0.05 was considered to be indicative of a statistically significant result.
